# KecNet: A Light Neural Network for Arrhythmia Classification Based on Knowledge Reinforcement

**DOI:** 10.1155/2021/6684954

**Published:** 2021-04-24

**Authors:** Peng Lu, Yang Gao, Hao Xi, Yabin Zhang, Chao Gao, Bing Zhou, Hongpo Zhang, Liwei Chen, Xiaobo Mao

**Affiliations:** ^1^School of Information Engineering. Zhengzhou University, Zhengzhou 450001, China; ^2^Department of Automation, School of Electrical Engineering, Zhengzhou University, Zhengzhou 450001, China; ^3^Collaborative Innovation Center of Internet Healthcare, Zhengzhou 450052, China; ^4^Department of Rehabilitation Medicine, Children's Hospital, Zhengzhou University, Henan Children's Hospital, Zhengzhou 450018, China; ^5^State Key Laboratory of Mathematical Engineering and Advanced Computing, Zhengzhou 450001, China

## Abstract

Acquiring electrocardiographic (ECG) signals and performing arrhythmia classification in mobile device scenarios have the advantages of short response time, almost no network bandwidth consumption, and human resource savings. In recent years, deep neural networks have become a popular method to efficiently and accurately simulate nonlinear patterns of ECG data in a data-driven manner but require more resources. Therefore, it is crucial to design deep learning (DL) algorithms that are more suitable for resource-constrained mobile devices. In this paper, KecNet, a lightweight neural network construction scheme based on domain knowledge, is proposed to model ECG data by effectively leveraging signal analysis and medical knowledge. To evaluate the performance of KecNet, we use the Association for the Advancement of Medical Instrumentation (AAMI) protocol and the MIT-BIH arrhythmia database to classify five arrhythmia categories. The result shows that the ACC, SEN, and PRE achieve 99.31%, 99.45%, and 98.78%, respectively. In addition, it also possesses high robustness to noisy environments, low memory usage, and physical interpretability advantages. Benefiting from these advantages, KecNet can be applied in practice, especially wearable and lightweight mobile devices for arrhythmia classification.

## 1. Introduction

Arrhythmias are the most common cardiovascular disease and the leading cause of stroke and sudden cardiac death. Electrocardiogram (ECG) is a common tool for detecting arrhythmias because of its noninvasive and easy-to-perform nature. Because of the randomness of arrhythmia onset, it is necessary for patients to be monitored for a long period, causing the difficulty of processing the resulting large number of ECG data. A computer-aided arrhythmia diagnosis system running on mobile devices can reduce labor and responding time, thereby improving the efficiency of daily arrhythmia classification [1, 2].

Most of the existing algorithms for automatic ECG recognition of arrhythmia are based on the assessment of morphological features of single or fewer heartbeats. Due to individual differences, the methods based on short-term features are prone to errors. In addition, numerous clinical studies have demonstrated the importance of long-term rhythm features for detecting arrhythmia associated with many diseases such as tachycardia, atrial fibrillation, and premature beats. This motivated us to study a new solution to arrhythmia classification based on a long period of continuous ECG signals.

In recent years, ECG classification algorithms based on deep neural networks (DNNs) have demonstrated excellent performance. DNNs use raw ECG data as input to build an end-to-end model for feature learning and classification to achieve comparable accuracy for both noise-free data and noisy data. Among them, convolutional neural networks (CNNs) [3–5] and recurrent neural networks (RNNs) [6–8] are the two most frequently used classes of neural nets.

One of the major problems with DNNs is that the performance of the algorithm is highly dependent on the network's scale. Most of the current mobile devices are still limited in both computation power and memory capacity and thus unfit for existing DNN approaches. Therefore, it is crucial to building lightweight networks that can operate in resource-constrained environments. Currently, there exist two main approaches to build lightweight networks. (1) The first approach uses network pruning techniques [9] or knowledge distillation [10] to achieve model compression and inference acceleration by removing redundant structures and parameters. Because the accuracy-focused models contain strategies that help overcome various problems encountered during training, such as overfitting, it is difficult to scale such a model down sufficiently without sacrificing accuracy. (2) The second approach designs models specifically for resource-constrained environments. Such efficiency-focused models include MobileNet [11], ShuffleNet [12], and LiteNet [13]. This approach sacrifices model performance to achieve a more efficient network structure [14].

On the other hand, DNNs fail to take advantage of useful features established by cardiologists in the past decades; therefore, we need an immense amount of high-quality labeled data to learn the potential causal relations between ECG data and diseases. Such data are often difficult to obtain and expensive. Therefore, only limited data sets can be used for model training. Because of the inevitable contamination by various noises in practical application, models built on limited data sets may be at risk of low robustness. Therefore, it is still a challenge to design a lightweight and robust DL algorithm for resource-constrained mobile devices.

Recent studies have shown that neural networks approximate the mapping between input and output data. It is believed that the mapping can be simplified by using domain knowledge or explicit features of the raw data, and the network presentation capability and scale can be optimized accordingly [15]. In ECG signals, the information reflecting diseases corresponds to a certain frequency range. Based on these findings, a lightweight DL architecture named KecNet is implemented to recognize 10-second ECG signal fragments in this study. 10 seconds is the typical duration of the central rhythm strip acquisition on a routine 12-lead ECG [16]. The developed scheme includes the following features. Firstly, a CNN network containing a customized filter bank is designed based on digital signal processing to precisely fit each application. By imposing constraints on filter shapes, it effectively extracts components in a specific frequency range from complex signals. Because the filter design is incorporated with the knowledge of digital signal processing, the proposed approach performs more physical interpretability than the conventional CNN [17]. It is also much more robust against noisy environments and less consuming in terms of implementation cost. Secondly, the long-duration correlation features of the ECG fragments are represented symbolically based on the clinical knowledge, which is used as an additional parameter to further constrain the decision-making process and improve the performance of the model. We evaluate the performance of the proposed KecNet on the MIT-BIH arrhythmia database. According to the Association for the Advancement of Medical Instrumentation (AAMI) protocol [18], heartbeat types in the MIT-BIH database [19] can be sorted into 5 main classes: normal beat (N), supraventricular ectopic beat (S), ventricular ectopic beat (V), fusion beat (F), and unknown (Q). Experimental results show that the proposed method has better performance and robustness for the arrhythmia classification task.

## 2. Problem Definition

Most deep learning-based approaches to arrhythmias adopt the form of supervised learning to learn mapping functions. Mathematically, we denote an ECG data set containing N samples as *X*={(*x*^(*i*)^, *y*^(*i*)^)*|i* ∈ *N*}, where *x*^(*i*)^ is the *i*^th^ sample and *y*^(*i*)^ is the label. The procedure for the neural network learning about data can be viewed as a parameter optimization problem, as formulated in

(1) $$\begin{array}{c} \theta_{f}^{*}=\underset{\theta_{f}}{\text{argmin}}\sum_{i\in N}L\left(f\left(x_{*}^{\left(i\right)},\theta_{f}\right),y^{\left(i\right)}\right), \end{array}$$

where *f*(*∗*, *θ*_*f*_) is the function that we will design to simulate the mapping between data and labels and *θ*_*f*_ is the parameters associated with the mapping *f*(*∗*). *L*(*∗*) is a loss function describing the loss of assigning a prediction category for a sample *x*^(*i*)^ with label *y*^(*i*)^.

Due to the complexity of arrhythmia pathology, the conventional approach for improving the performance of classification models is to increase the number of layers of the network and enhance the representation capability of the model by adding nonlinear operations. However, this poses three problems. Firstly, the increase in the number of layers causes the increase of parameters in the network, which intensifies the storage difficulty and computational complexity of the model. Secondly, the increase in model depth may cause the risk of vanishing gradient [20], resulting in the inability to effectively update the parameters of the shallow convolution kernel. Thirdly, more training data is needed to prevent overfitting of the model [21].

Based on the above analysis, our goal is to achieve better classification performance using shallow networks without increasing the model complexity and training data. To achieve this goal, we try to incorporate relevant domain knowledge into the design process of convolutional neural networks, mainly including using the amplitude-frequency characteristics of bandpass filters to filter the noise in ECG signals to improve the model's ability to grasp valid information, and extracting dominant features of ECG data as additional parameters to prevent the loss of temporal information due to the pooling mechanism of CNN.

## 3. Methods

**Figure 1:**
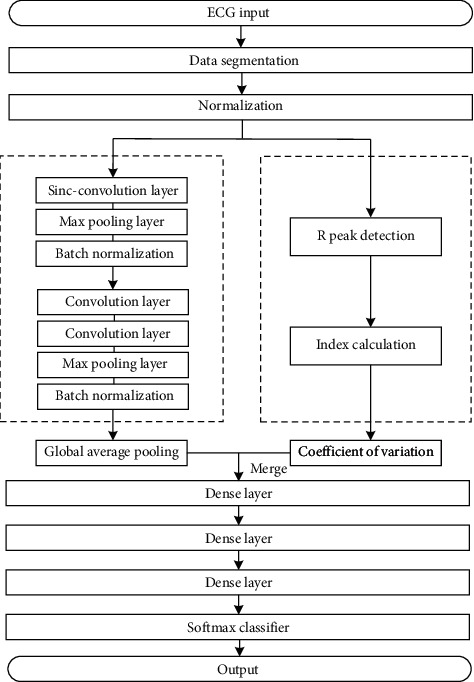
Workflow of the proposed method.

The workflow of the proposed method is shown in Figure 1. First, the data is segmented and normalized. The segmented data are fed into the KecNet model, which contains a CNN structure with a customized convolutional layer and a symbolic parameter extraction structure in the feature extraction part of the model. Finally, the fused feature vectors are fed into the softmax classifier for classification.

### 3.1. Sinc-Convolution Layer

The ECG signal is a mixture of electrical activity from various parts of the myocardium. Depending on the quality of the data, it may also include multiple types of noise, such as baseline drift, motion artifacts, and electromyographic interference [22]. The filter information learned by traditional CNNs usually contains a mixture of noise and multiband modes, which reduces the representation capability and readability of the ECG signal [17].

In order to overcome this defect, it is very important to optimize the first convolution layer of CNN because this layer directly processes the original ECG samples containing rich underlying information and helps the subsequent convolution layer to perform complex nonlinear representation of data. This study introduces the Sinc-convolution layer, a structure based on the interpretable CNN developed by Ravanelli and Bengio for speech recognition [17]. The core idea of this structure is based on parameterized cardinal-sine (Sinc) functions for bandpass filter design. While the conventional CNN learns all the parameters of the filters, the Sinc-convolution defines a tunable filter bank *g* with clear physical explanation in advance to replace the filter in traditional CNN, as formulated in

(2) $$\begin{array}{c} s\left[n\right]=x\left[n\right]*g\left[n,\theta_{g}\right], \end{array}$$

where *θ*_*g*_ is the parameter to be learned. For ECG signal analysis, the bandpass filter is an effective choice for designing tunable filter banks [23], because the time domain signal is divided into different subspaces by frequency band transformation so that the filter can be activated by the information of specific frequency band to achieve more effective and reliable filtering.

In the frequency domain, the amplitude of a universal bandpass filter can be written as the difference between two rectangular filters. After returning to the time domain using the Fourier transform [23], the function *g* becomes as

(3) $$\begin{array}{c} g\left(n,f_{L},f_{H}\right)=2f_{H}\sin\;c\left(2\pi f_{H}n\right)-2f_{L}\sin\;c\left(2\pi f_{L}n\right), \end{array}$$

where *f*_*L*_ and *f*_*H*_ are the learned low and high cut-off frequencies, and sin  *c*(*x*)=sin(*x*)/*x*.

To mitigate the spectral leakage, a popular solution is windowing by multiplying the truncated function *g* with a window function:

(4) $$\begin{array}{c} g_{\varpi }\left(n,f_{H},f_{L}\right)=g\left(n,f_{H},f_{L}\right)*\varpi \left(n\right). \end{array}$$

In this work, we use the Hamming [15] window as follows:

(5) $$\begin{array}{c} \varpi \left(n\right)=0.54-0.46\;\cos\left(\frac{2\pi n}{L}\right). \end{array}$$

**Figure 2:**
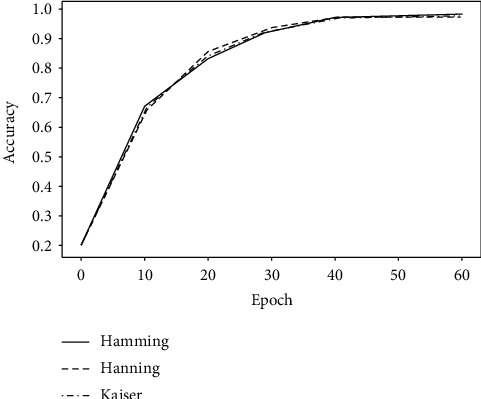
Comparison of different window functions.

In fact, as shown in Figure 2, similar results are obtained when training arrhythmia classification models, regardless of whether the window function type is Hamming, Hanning [16], or Kaiser [22]. In addition, since filter *g* is symmetric, the computational efficiency can be improved with one side of the filter inheriting the results from the other side.

The experimental results in Section 5.3 show that the Sinc-convolution layer is more selective in frequency response compared to the CNN. Because the filter effectively extracts components from complex signals over a specific frequency range, it improves the model's robustness and readability [24]. After the Sinc filter self-adaptively classifies the frequency band of the raw ECG data, the standard CNN structure is used to extract the time domain features.

### 3.2. Symbolic Representation of Rhythmic Features

When analyzing the discrete-time series of the data, converting sequences into symbols with practical meaning is a common method to simplify the analysis process [25]. In fact, some quantitative features based on clinical knowledge (e.g., heart rate variability [26], RR interval [27], etc.) are considered to be more relevant to the underlying pathological mechanisms. Among them, the coefficient of variation (CV) describes the degree of dispersion of the RR interval and is commonly used to measure the regularity of the RR interval [26]. Since the R-peak is the most obvious waveform in the ECG, features based on the RR interval have stronger noise immunity and are one of the most important features for analyzing the rhythm variation [28].

The model is optimized based on the above findings. In addition to the CNN structure designed to extract spatial morphological features, the CVs were added to the network as symbolic representations of rhythm features. ECG fragments with anomalous rhythms are identified more easily. The computational procedure is as follows/

**Step 1 .**

Detect the R-peak of ECG fragment *x*^(*i*)^ based on the Pan-Tompkins algorithm [29] to obtain the sequence of R-peak positions *R*^(*i*)^={*r*_1_^(*i*)^, *r*_2_^(*i*)^,…, *r*_*j*_^(*i*)^}.

**Step 2 .**

Calculate the RR interval series *T*^(*i*)^={*t*_1_^(*i*)^, *t*_2_^(*i*)^,…, *t*_*n*_^(*i*)^}.

**Step 3 .**

Calculate the mean value $\bar{t}=\sum_{i=1}^{n}t_{i}/n$ and standard deviation $\sigma =\sqrt{\sum_{i=1}^{n}\left(t_{i}-\bar{t}\right)/n}$ of the sequence *T*^(*i*)^.

**Step 4 .**

Calculate the coefficient of variation $CV^{\left(i\right)}=\left(\sigma /\bar{t}\right)$.

### 3.3. The Architecture of the Network

The proposed KecNet is based on a 1D convolutional neural network, including one Sinc-convolution layer, two standard convolution layers, three pooling layers, two batch standardization layers, three dropout layers, one global average pooling layer, and three dense layers.  Table 1 summarizes the basic structure of KecNet. The key characteristics of different layers in basic KecNet are detailed as follows:

- Sinc-convolution layer: The length of the filter significantly affects the classification accuracy. With the increase of filter length, the accuracy is improved. Through experiments, the length of convolution kernel L = 251 is selected. It should be noted that since a Sinc filter has only two parameters, no matter how long the filter length is selected, the parameters will not increase
- Standard convolution layer: Two 1D convolution layers are added after the Sinc-convolution layer and convolve with a filter size of 5. All convolution layers (including Sinc-convolution layer) adopt ReLU activation function [30]
- Max-pooling layer: KecNet performs a max-pooling operation after the Sinc-convolution layer and two standard convolution layers. The max-pooling operation reduces the computation cost between the convolution layers while achieving translation invariance of the neural network
- Dropout [31] layer: The dropout layer reduces the complex coadaptation relationships between neurons by randomly dropping a fraction of the network nodes and overcomes the overfitting problem at the same time. We build the dropout layer after each of the two groups of pooling layers, with a ratio of 0.2. We also add a dropout layer after the first full connection layer, with a ratio of 0.3
- Batch Normalization (BN) [32] layer: The BN technique ensures the validity of the gradient by adjusting the distribution of the output data, smoothing the loss plane, and speeding up the convergence of the network. We apply the BN layer after the two dropout layers
- Global Average Pooling (GAP) [33] layer: GAP averages the values of all elements of the feature map to reduce the number of parameters. We use the GAP layer before the dense layer. Also, the rhythm feature notation described in Section 3.2 is added to the GAP layer to maximize the impact of this factor on the network
- Dense layer: Two fully connected layers are used in the basic KecNet. The first layer consists of 16 units. The second layer consists of 8 units. After dense layers, the softmax function is used as a classifier to predict five classes

## 4. Experimental Setup

### 4.1. Materials and Preprocessing

We used the MIT-BIH arrhythmia database to evaluate the performance of the proposed method. The database contains 48 dynamic ECG recordings, each with 30-minute long, 360 Hz sampling rate, 11-bit resolution, and ±10 mv dynamic range. Each recording contains two lead configurations (usually MLII and V1). Lead II is commonly used for wearable single-lead ECG sensors. According to the AAMI protocol, we merged the original 18 categories of heartbeat types in the MIT-BIH database into 5 major categories. The heartbeat classes' mapping between the AAMI protocol and the MIT-BIH database is shown in Table 2.

After data merging, a sliding window with length *M* = 3600 is set to segment the data. Because the data sampling rate is 360 Hz, it is equivalent to using about 10 s of data as input. To overcome the data imbalance problem, the data were synthesized by translating the start point for small-size data. After the enhancement, each category has 55000 samples. Because each signal in the MIT-BIH dataset is labeled with a disease class accurate to the second, the class with the largest percentage is used as the label for that ECG segment. Finally, the ECG segments are normalized by *Z*-score to solve the problems of amplitude scaling and eliminate the offset effect.

**Figure 3:**
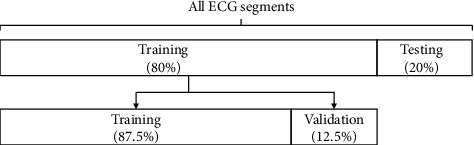
The percentage of ECG segments used for training and test.

The data set is divided into two mutually exclusive sets: training set (80%) and testing set (20%), and 12.5% of the data in the training set is used as the validation set. To ensure the consistency of the data distribution, each class is randomly sampled separately according to the proportion of the data set. The data set division is shown in Figure 3.

### 4.2. Evaluation Criteria

The following indicators are used to evaluate the proposed method:

(i) Accuracy (ACC): ACC is an overall measure of the correctness of arrhythmia classification results relative to the entire sample.
  (6) $$\begin{array}{c} \text{ACC}=\frac{\text{TP}+\text{TN}}{\text{TP}+\text{TN}+\text{FP}+\text{FN}}. \end{array}$$
(ii) Sensitivity (SEN): SEN indicates the proportion of samples that were correctly predicted in all the samples that were truly positive. For disease classification, sensitivity is a very important criterion, with the higher sensitivity indicating the lower miss rate of the model.
  (7) $$\begin{array}{c} \text{ SEN}=\frac{\text{TP}}{\text{TP}+\text{FN}}. \end{array}$$
(iii) Precision (PRE): PRE indicates the proportion of samples that were true positive in all the predicted positive samples.
  (8) $$\begin{array}{c} \text{PRE}=\frac{\text{TP}}{\text{TP}+\text{FP}}. \end{array}$$
(iv) Parameter Count (PC): In DL, PC represents the model size and the number of unit connections between layers (computational cost). It is an important factor that affects the computation complexity of DL algorithms. The lower the PC is, the lower the computation cost is and the less memory the model needs.

### 4.3. Hyperparameters Setting

**Figure 4:**
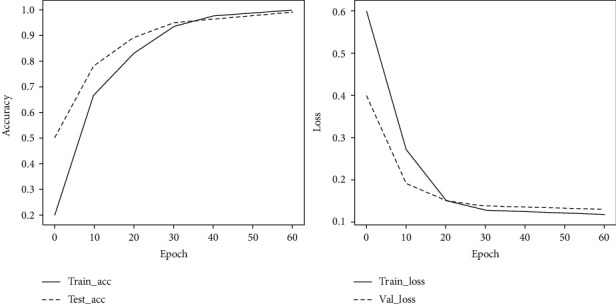
Changes in loss value and accuracy as epoch increases.

Figure 4 shows the accuracy and loss of the training and validation set of KecNet at each training phase. These curves indicate that the accuracy and loss of the model are stable, and the network basically converges after 60 epochs of training. Considering the model's validity and training efficiency, the epoch for the training model was set to 60. The hyperparameters are set by optimizing the model through trial and error, as shown in Table 3.

## 5. Results and Discussion

### 5.1. Analysis of Experimental Results of Model Performance

Table 4 shows the performance of the proposed method on the data set. A standard CNN architecture is used as the baseline model, and the length of the first layer convolution kernel is set to 32. It can be seen that the performance of Sinc convolution is better than that of standard convolution. Moreover, the parameters are reduced by about 80% compared with the same structure of CNN.

Table 5 shows the effect of the coefficients of rhythm variation on the model performance. The performance of the model is improved by 1–1.5%. However, the increase in time is negligible.

### 5.2. Implementation PC Reduction

For wearables and mobile devices, improving the accuracy of deep learning algorithms is not the only problem. Most devices have difficulties in deploying complex, high-performance models due to limited computing and storage capacity. Therefore, it is equally crucial to reduce the memory footprint of the model. PC represents the spatial complexity of the model, which is an important indicator of the model size and memory usage.

Having a low PC with guaranteed classification accuracy is an advantage of KecNet, because the Sinc-convolution structure greatly reduces the number of parameters in the first convolution layer. Since the low and high cut-off frequencies are the only parameters of the filter learned from ECG data, the number of parameters is only dependent on the number of filters. For example, for a layer with F filters of length *L*, a standard CNN contains *F* × *L* parameters, while a Sinc-convolution layer only requires *F* × 2. The parameter gain of the Sinc-convolution layer compared to CNN is shown in

(9) $$\begin{array}{c} \text{Gain}\left(\%\right)=\frac{{\text{PC}}_{\text{CNN}}-{\text{PC}}_{\text{KecNet}}}{{\text{PC}}_{\text{CNN}}}\times 100=\left(1-\frac{2}{L}\right)\times 100, \end{array}$$

where PC is the number of parameters. PC of the CNN increases with the increase of *L*, but the Sinc-convolution layer always remains the same. Accordingly, the longer the *L* is, the more gain the KecNet has in reducing PC in comparison with the standard CNN. For instance, the PC of KecNet is reduced by 80% compared to the standard CNN in Table 4.

In addition, we compare the classification performance of KecNet with the classical CNNs: GoogleNet [34], MobileNet, and SqueezeNet. The hyperparameters are set as in Table 3. Notably, MobileNet and SqueezeNet are also lightweight models and have been successfully deployed on resource-constrained mobile devices, such as cell phones, robots, and self-driving cars [35]. The experimental results are shown in Table 6. In terms of classification effect, KecNet outperforms SqueezeNet and MobileNets and is slightly lower than GoogleNet. However, in terms of PC, KecNet reduces the amount of PC by about 50% compared to SqueezeNet and MobileNet and by about 80% compared to GoogleNet.

### 5.3. Filter Analysis

**Figure 5:**
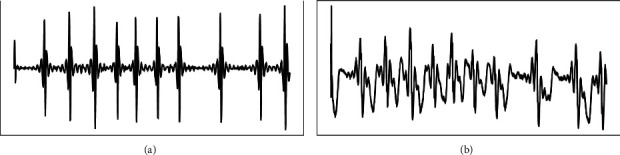
Examples of KecNet and CNN filters. (a) Temporal of KecNet filters. (b) Temporal of CNN filters.

For the feature mapping obtained from the first convolutional layer, KecNet has some advantages over the standard CNN in terms of interpretability and readability. This is mainly because the Sinc-convolutional structure used in the model is functionally equivalent to a bandpass filter, which learns parameters with a clear physical meaning, i.e., the high and low cut-off frequencies of the ECG signal [24].  Figure 5 shows examples of filters learned by KecNet (Figure 5(a)) and standard CNN (Figure 5(b)) using the MIT-BIH arrhythmia dataset. From the figure, it can be seen that the filtering result of standard CNN is noisy and not very readable. In contrast, the processing result of KecNet for ECG signal is significantly better than CNN and more regular.

**Figure 6:**
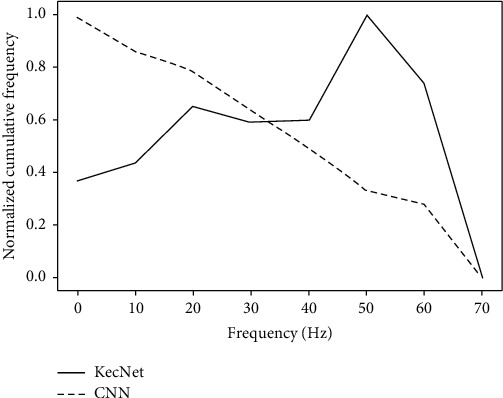
Cumulative frequency response comparison between KecNet and standard CNN.

In addition to comparing the effects, it is necessary to analyze which bands are covered by the filters.  Figure 6 shows the cumulative frequency response of the filters learned by KecNet and CNN on the arrhythmia classification task. The cumulative frequency response is obtained by normalizing the accumulation of all the filters in the convolution layer. By analyzing the cumulative frequency response, the importance of frequency-specific information for the arrhythmia classification task can be found, since the frequency bands with small normalized cumulative values are less important. It can be seen that the KecNet plot has two distinct peaks. The first one mainly concentrated in the 0 Hz–15 Hz range, which corresponds to the frequency domain range of the *P* and *T* waves in the ECG fragment. The second peak corresponds to the QRS wave in the ECG segment, with a frequency range of 20 Hz to 50 Hz. This result shows that KecNet has successfully adapted its characteristics to classify ECG signals. In contrast, the standard CNN does not exhibit a similarly meaningful pattern: its frequency response curve does not clearly represent the frequency band peaks of the corresponding bands. That is, the filters learned by KecNet are, on average, more selective than CNNs and thus can better capture the valid information in ECG signals.  Table 7 shows several examples of frequency bandwidths extracted with KecNet. It is worth mentioning that the ability to acquire the principal frequency component of the ECG signal enhances the robustness of the network.

### 5.4. Generalization and Robustness in Noisy Environment

Generally speaking, the data sets used to prove the effectiveness of the proposed method are usually collected under the same conditions. However, there are usually various interferences in real applications. In this case, the effectiveness of the automatic classification method will be greatly reduced since it is difficult to collect labeled data for the training purpose in all environments. In order to overcome this shortcoming, a robust method is needed. We discuss the robustness of KecNet in various environments by adding white noise to the data. All the models are trained with the original data sets. Then, they are tested with noisy signals.  Table 8 shows the variation in the accuracy of the models with the signal-to-noise ratio (SNR) from 0 to 60 dB.

It can be seen that the accuracy of all models is higher at higher SNR. However, as the SNR decreases, the accuracy (%) of the standard CNN model decreases significantly. The accuracy is below 80% when SNR is 20 dB or less. In contrast, the accuracy of KecNet is still higher than 98% and is stable. It is demonstrated that the proposed method is more robust than traditional CNN. The main reason for this result is due to the fact that the proposed method has a clear filter with a well-defined spectral shape at the first convolution layer, whereas the filter learned by the traditional CNN model is highly correlated with the training data and susceptible to noise interference.

## 6. Conclusion

In this work, we propose a lightweight end-to-end solution for resource-constrained mobile devices that leverages domain knowledge to optimize neural networks for arrhythmia classification. Firstly, we introduce a physically interpretable Sinc-convolutional layer to learn customized filters with clear bandwidths as features to improve the feature extraction ability of CNNs and reduce the number of parameters. Secondly, the rhythm variation coefficient is added to the network as a symbolic representation of time series to compensate for the difficulty of grasping long-duration correlation features in shallow CNNs and to improve network performance and clinical usability. We trained and tested on the MIT-BIH arrhythmia data set using raw ECG data. The ACC, SEN, and PRE reached as high as 99.31%, 99.45%, and 98.78%, respectively. In addition, neural network size is reduced, and robustness to noise was increased.

In the future, we will collect and annotate ECG recordings from real patients and study the classification of more different types of diseases. In addition, multilead ECG recordings will be used for training models. On the clinical side, we will develop an ECG system that can be deployed on wearable medical devices and low-cost ECG devices, test, and improve its performance.

## Figures and Tables

**Table 1:** Model parameters.

Layer		Feature map element no.	Kernel size	Stride
Sinc module	Sinc-Conv1D	32	251	1
	Max-pooling	—	2	2
	Dropout	—	—	—
	BN	—	—	—

Conv module	Conv1D	16	5	1
		16	5	1
	Max-pooling	—	2	2
	Dropout	—	—	—
	BN	—	—	—

GAP	16 + 1	—	—	—
Dense	16	—	—	—
Dropout	—	—	—	—
Dense	8		—	—
Softmax	5	—	—	—

**Table 2:** Heartbeat classes' mapping between the AAMI protocol and the MIT-BIH database.

AAMI classes	MIT-BIH classes
Normal beat (N)	NOR, NE, AE, LBBB, RBBB
Supraventricular ectopic beat (S)	AP, APB, APC, NP, SP
Ventricular ectopic beat (V)	VF, VE, PVC
Fusion beat (F)	F
Unknown (Q)	UN, FPN, P

**Table 3:** Hyperparameters of KecNet.

Batch size	Epochs	Optimizer	Beta_1	Beta_2	Lr
128	60	Adam	0.9	0.999	0.0003

**Table 4:** Performance comparison between standard convolution and Sinc-convolution.

Convolution type	ACC (%)	SEN (%)	PRE (%)	PC	Test (ms)
Standard	96.34	95.78	96.53	1226	73.2
Sinc	98.64	98.23	97.98	266	49.4

**Table 5:** Comparison of models performance with the coefficient of variation.

CV	ACC (%)	SEN (%)	PRE (%)	PC	Test (ms)
Without	98.64	98.23	97.98	266	49.4
With	99.31	99.45	98.78	267	50.1

**Table 6:** Comparison of model performance.

Model	ACC (%)	SEN (%)	PRE (%)	PC
GoogleNet	99.42	99.51	99.07	1326
MobileNet	97.57	97.29	97.00	580
SqueezeNet	97.69	97.23	97.13	562
KecNet	99.31	99.45	98.78	267

**Table 7:** Examples of the frequency bandwidths extracted by KecNet.

*f*_*L*_	0.7	1.2	4.4	10.7	10.1	23.5

*f*_*H*_	8.5	7.4	11.7	51.4	43	52.1

**Table 8:** Comparison of model performance under different SNRs.

(dB)	CNN	KecNet	KecNet + CV
10	78.56	97.33	98.39
20	79.88	97.72	98.26
30	85.78	98.12	98.64
40	89.21	98.98	98.77
60	96.22	98.53	99.26

## Data Availability

All data sets used to support the findings of this study are included within the article. All data sets used to support the findings of this study were supplied by the publicly available MIT-BIH database from the Massachusetts Institute of Technology. The URL to access the data is https://www.physionet.org/content/mitdb/1.0.0/
